# Language as a cultural trigger: a study of pointing gestures in Bengali-English bilinguals

**DOI:** 10.3389/fpsyg.2026.1763681

**Published:** 2026-03-25

**Authors:** Xuan Wang

**Affiliations:** School of International Education (Ural), North China University of Water Resources and Electric Power, Zhengzhou, Henan Province, China

**Keywords:** bilinguals, pointing gestures, cultural frame switching, high-versus low-context, human cognition

## Abstract

Although gesture research in bilingualism has predominantly focused on iconic and metaphoric gestures, pointing behaviors remain understudied. This study investigates whether Bengali-English bilinguals adjust pointing when switching languages, given the distinct pointing norms in these two linguistic contexts. Grounded in Cultural Frame Switching (CFS) theory, it examines whether language alone triggers measurable shifts in the frequency and form of pointing gestures. Thirty-one Bengali-English bilingual university students participated in two rounds of semi-structured interviews conducted in Bengali and English, with 1 month interval in-between. Video recordings were transcribed and coded for both manual and non-manual (head, lips, eyes) pointing gestures. Results showed that participants produced significantly more manual pointing in English than in Bengali (*p* < 0.05), while non-manual pointing was significantly more frequent in Bengali than in English (*p* < 0.05). These findings provide quantitative evidence that language functions as a cultural prime, recalibrating embodied behavior: English activates a low-context frame demanding explicit reference, whereas Bengali activates a high-context frame emphasizing politeness and indirectness. By demonstrating how bilinguals switch gestural strategies in response to linguistic cues, this study advances CFS theory and contributes to a deeper understanding of the embodied nature of human cognition. It also offers practical implications for intercultural communication training and AI gesture recognition systems.

## 1 Introduction

While current bilingual research mainly charts the development of spoken and written language, an increasing body of work has also examined gestures (Tunagür et al., 2021; Nicoladis and Smithson, 2022; Nicoladis and Gao, 2022; Nicoladis et al., 2024; Hoot, 2025). Comparatively, bilingual gestures have not been as widely studied as spoken and written language. Gestures act as a fundamental multimodal resource in bilingual language processing, operating not as a supplementary element but as a constitutive facet of speech itself, with distinct cognitive and communicative purposes (Arslan et al., 2023; Khatin-Zadeh et al., 2023; Khatin-Zadeh et al., 2025a). However, within this small pool of research, scholarly attention leans overwhelmingly toward iconic and metaphoric gestures (Graziano et al., 2020; Wermelinger et al., 2020). This inclination is reasonable; the visual-conceptual correspondence these gestures hold with their referents delivers potent multimodal reinforcement, thereby easing second language (L2) acquisition (Macedonia, 2025). A sustained emphasis on these categories, however, risks undervaluing the important and potentially unique roles that pointing gestures play in bilingual interactions. These gestures seem to control discourse organization, externalize spatial references, and fill in lexical gaps in a speaker’s less proficient language (Azar et al., 2020a).

Pointing is widely regarded as the earliest gesture used to communicate, bridging pre-verbal infants’ world and adult language (Morgenstern, 2022); their universal yet culturally shaped use makes them an ideal window into how bilinguals adjust attention, common ground and politeness across language-specific ecologies (Cartmill, 2022). Because pointing synchronizes social-cognitive and linguistic sub-systems within a single, time-locked motion (Krivokapic et al., 2016), subtle shifts in its form or frequency can disclose real-time reorganization of the bilingual mind that solely verbal measures might miss. As such, this paper explores the pointing gestures in bilingual speakers.

Pointing involves directing a body part toward a specific target, such as a person, object, or place, in order to create a temporal and spatial connection that will direct others’ attention to that target (Cooperrider and Mesh, 2022). As a fundamental pragmatic mechanism, it aligns interlocutors’ focus, anchors language in physical context, and facilitates mutual comprehension (Morgenstern and Goldin-Meadow, 2022). Importantly, pointing is not confined to the hand, speakers also recruit head movements, and facial actions such as lip protrusion or nose wrinkling (Cooperrider and Mesh, 2022; Li, 2024).

The present study focuses on Bengali-English bilinguals for they demonstrate noticeable differences in the form of their pointing gestures. Prior work indicates that pointing in Bengali interaction carries social meaning: an open-hand gesture may signal politeness when addressing elders, while a single-finger point can be perceived as disrespectful (Uddin, 2019). Furthermore, Bengali speakers commonly employ non-manual pointing, using their eyes, chin, or head, a practice that aligns with cultural preferences for indirect and nuanced communication (Arif, 2011). In contrast, American English speakers often rely almost exclusively on index-finger pointing, overlooking non-manual alternatives (Cooperrider et al., 2018). Besides the pointing form variances, the Bengali-English bilinguals may also adjust their gesture frequency depending on whether they are speaking Bengali or English, and on the communicative situation (Lopez-Ozieblo, 2024; Azar et al., 2020a). While the aforementioned studies have been instrumental in mapping the gestural landscapes of monolingual groups, the territory of bilingual individuals remains notably uncharted (Uddin, 2019; Cooperrider et al., 2021). It is also unknown whether individuals who navigate two linguistic worlds maintain two distinct gestural systems as they switch languages. To close this gap, the current study examines a group of Bengali English bilinguals and investigates how their pointing gestures vary depending on the linguistic contexts.

## 2 Literature review

### 2.1 Bilingualism and pointing gestures

Research has focused on the cultural transferability of gestures in bilingual contexts and its influence on linguistic representation. The early work of Cavicchio and Kita (2013) investigated whether English-Italian early bilinguals transfer gestural patterns between languages by testing 30 university students (10 English monolinguals, 10 Italian monolinguals, and 10 matched bilinguals with equal proficiency) who described motion-event videos to native listeners. They measured gesture rate (gestures per word) and salience (central vs. peripheral gesture space), finding that Italian speakers gestured more frequently and saliently than English speakers. Crucially, bilinguals switched these parameters according to the language spoken without transfer effects, though they produced overall more salient gestures than monolinguals. This foundational research emphasizes that bilingual-gestural interactions are not linguistic strategies alone, but dynamic adaptive processes shaped by the interplay of cognitive and cultural factors.

Recent studies have demonstrated that the strategies bilingual individuals employ to manage the mental demands of switching between two languages through gesture use. For instance, Avcı et al. (2022) found that adult Turkish-English bilinguals produced more representational gestures in their second language (English) than in their native Turkish, and this increased gesture use was associated with reduced disfluency rates, suggesting that gestures facilitate lexical retrieval under higher cognitive load. Likewise, Cienki and Iriskhanova (2020) demonstrated that professional interpreters performing simultaneous translation under extreme time pressure relied heavily on beat gestures, which helped them maintain narrative coherence and manage the cognitive demands of real-time language switching. Therefore, gesture production may decrease cognitive load and facilitate cognitive processes (Kita et al., 2017).

In addition, deictic gestures have also been documented among varied populations, including older adults (Arslan and Göksun, 2022) and bilingual individuals (Azar et al., 2020b), implying that pointing at abstract referents may lighten the cognitive burden for these speakers. This suggests that gestures serve as “simulators” that externalize spatial-semantic features under dual-language pressure, freeing working memory from lexical search and thereby smoothing following speech encoding.

As for the gesture frequency of the bilinguals, several researchers link gesture rates to the second language (L2) proficiency. A congruent view is advanced by Aziz and Nicoladis (2019) and Arslan et al. (2024), who both contend that bilinguals’ gesture rates are tightly coupled with L2 proficiency rather than being purely compensatory. For instance, Aziz and Nicoladis (2019) noted that English-French bilinguals living in an English-speaking environment gestured more frequently in their less proficient French, linking this rise to their lower command of the language. Arslan et al. (2024) corroborate this proficiency-dependency by demonstrating that gestures surface primarily during disfluencies and co-occur with successful error detection, indicating that even high-proficiency bilinguals rely on manual mimicry to regulate L2 output. Thus, while one interpretation views gesturing as a way to compensate for language gaps, the other reinterpret it as a self-regulating mechanism used by bilinguals regardless of proficiency.

Other research shows that bilinguals may increase gesture use to reduce cognitive load, thereby organizing speech and facilitating language processing when managing two languages (Nicoladis et al., 2009). Supporting this idea, Nicoladis et al. (2019) found that bilinguals who speak Mandarin, Hindi, French or Spanish tend to use slightly more gestures when speaking their second language (English). More recent study by Arslan et al. (2024) revealed that Turkish-English bilinguals may use more gestures in the L2 due to higher cognitive load, especially during moments of speech disfluency.

Gesture rates are also influenced by task demands and input. Gámez et al. (2025) showed that young Spanish-English bilinguals produced more pointing gestures in the language with richer input, which in turn predicted later vocabulary growth. Data on adults further complicates the picture: once speech length is controlled for, gesture frequency correlates more with personality traits such as neuroticism than with L2 proficiency (Lopez-Ozieblo, 2024). Khodadadi et al. (2024) examined gesture production among Farsi-English bilinguals across two dimensions: task type (storytelling vs. language learning history) and language (Farsi vs. English). The results showed that participants produced more representational gestures in storytelling than in the language history task across both languages, and more beat gestures in English than Farsi, while there was no difference in the representational gesture rates across both languages.

### 2.2 Cultural frame switching

While the studies above focus on the cognitive and linguistic determinants of gesture, the phenomenon of Cultural Frame Switching (CFS) provides a broader framework for understanding how cultural schemas, triggered by context, can shape behavior, gesture in particular.

Cultural Frame Switching (CFS) describes the mental process where individuals with multiple cultural backgrounds adjust their thinking, behavior, and cognitive styles based on situational signals (Hong et al., 2000). Rooted in the Dynamic Constructivist Approach, this theory suggests culture is not static but a network of knowledge that can be switched on or off depending on the situation (Hong et al., 2000). In other words, specific cultural cues—such as language, symbols, or images—can trigger the activation of related cultural schemas, which then shape individuals’ self-construals, social judgments, and behaviors (Hong et al., 2000). The mechanism of CFS is typically explained through the process of priming. Empirical research demonstrates that cultural triggers increase the prominence of linked ideas and values among bicultural individuals. In one classic experiment, participants displayed more collectivist views after being exposed to Chinese icons but showed greater individualism after seeing American symbols (Hong et al., 2000; Mok and Morris, 2009). This shows how a single individual may rely on different cultural lenses depending on the context.

Cultural Frame Switching (CFS) in bilingualism reveals language as a potent activator of culturally embedded cognitive frameworks, enabling bilinguals to unconsciously shift between distinct cultural identities, adjusting their speech, conduct, and communication styles accordingly (Cortés, 2021; Liu et al., 2021). Empirical evidence demonstrates that such language switching activates culturally situated memory systems, producing measurable differences in moral judgment and self-expression across cultural contexts (Mishra, 2018; Wong and Ng, 2018). Extending beyond these behavioral effects, recent research has investigated CFS impacts on self-construal (Yim and Clément, 2021), learning strategies (Martinez, 2023), and psychological well-being (Garcha et al., 2025). However, emerging work challenges the assumption that frame switching is exclusively covert, showing that bodily cues such as dance posture can overtly instantiate these shifts (Pope, 2020). This somatic manifestation implies that if cultural frames indeed permeate the body, then language as a priming mechanism should likewise sculpt immediately observable movement patterns.

The present study tests this possibility by asking whether speaking Bengali versus English alone is sufficient to trigger measurable changes in manual and non-manual pointing during spontaneous discourse, providing the bodily evidence that language can act as a stand-alone cultural cue and extending CFS from internal cognition to embodied interaction.

### 2.3 Hypothesis development

Based on Cultural Frame Switching (CFS) theory, this study posits that language alone acts as a situational cue that unconsciously activates culturally embedded communicative schemas, which in turn shape visible bodily behaviors. When Bengali-English bilinguals switch languages, they are priming distinct cultural frames, carrying different norms for reference, politeness, and social harmony (Uddin, 2019). Hall’s (1976) distinction between high-context and low-context cultures provides a compatible perspective: high-context cultures depend on shared background and implicit cues, whereas low-context cultures require explicit verbal messages. The following four hypotheses jointly test whether this language-driven frame switching exhibits in both the frequency and form of pointing gestures.

*H1*: The frequency of pointing gestures is lower in Bengali than in English.

Speaking Bengali triggers a high-context cultural frame that highlights implicitness and shared understanding, reducing the need for explicit spatial marking (Uddin, 2019; Hall, 1976). Conversely, speaking English activates a low-context frame that prioritizes explicit specification to ensure comprehension; pointing gestures thus serve as essential tools to complement speech and reduce cognitive load in this less dominant context.

*H2*: In the Bengali context, non-manual pointing is preferred over manual pointing.

Politeness and respect serve as a core schema within the high-context Bengali frame; direct index-finger pointing is coded as confrontational, while non-manual pointing like head tilts or lip protrusions suggest indirectness (Uddin, 2019; Arif, 2011). When the Bengali cultural frame is primed, these social norms should bias the gestural system toward non-manual pointing that corresponds to the frame’s emphasis on face-saving and subtlety, even though such gestures are less visually salient.

*H3*: In the English context, the bilinguals prefer manual pointing.

The English low-context frame prioritizes directness and precision in communication; highly visible and spatially accurate, manual pointing aligns with these values and is accepted as culturally appropriate (Hall, 1976). Once the frame is activated, the hand becomes the default pointing tool since it best meets the frame’s need for explicit reference. This preference should display a higher number of manual points as well as a higher proportion of manual over non-manual pointing.

Collectively, these hypotheses investigate whether bilinguals dynamically recalibrate their pointing system (both in frequency and form) as they switch languages. If supported, the pattern would demonstrate that language alone acts as a cultural prime, producing distinct high- versus low-context interactional styles in real-time embodied behavior.

## 3 Methods

This study is part of a larger project that investigates the frequencies of various gesture types (referring to Table 1). However, gestures like iconic, metaphoric, and beat lie outside the focus of the present paper, therefore they will not be analyzed in detail.

**Table 1:** Paired samples test for the hand gestures (*N* = 30).

Pair	Variable	Paired differences					*t*	df	Sig. (2-tailed)
		Mean	Std. deviation	Std. error mean	95% confidence interval of the difference				
					Lower	Upper			
Pair 1	Total number of hand gestures	−18.50000	51.82514	9.46193	−37.85182	0.85182	−1.955	29	0.060
Pair 2	Iconic number	2.83333	7.81724	1.42723	−0.08567	5.75234	1.985	29	0.057
Pair 3	Metaphoric number	−0.50000	6.30134	1.15046	−2.85296	1.85296	−0.435	29	0.667
Pair 4	Deictic number	−6.80000	13.77754	2.51542	−11.94462	−1.65538	−2.703	29	0.011
Pair 5	Beats number	−13.50000	32.65045	5.96113	−25.69188	−1.30812	−2.265	29	0.031

### 3.1 Participants

Thirty-one students (five women and twenty-six males, ages about 20–29) participated, and one of them hosted the interviews. Flyers were posted in English-medium courses at a central Chinese polytechnic university, inviting degree-seekers from Bangladesh, and interested students emailed the research assistant. They should be native speakers of Bengali and fluent in English. Participants were also required to be between 18 and 30 years old and to have been residing in China for a minimum of 3 months. For those who have speech or hearing impairment are not included. The interviewer was selected on his communication experience and language fluency of both English and Bengali. He is the monitor of the class and has much contact with the participants, and also speaks both English and Bengali well. Moreover, he is blind to the purpose and hypotheses of the study. The study received approval letter from the Institutional Review Board from School of International Education (2024-SIE/IRB-0926), and written informed consent for participation in this study was provided by the participants. They were given a small gift in return and could withdraw anytime.

Each participant is fluent in English and speaks Bengali as their first language. This is because English is widely used in Bangladesh, particularly in the domains of education and administration (Rahman, 2014; Hossain, 2016). Actually, the country’s primary language of instruction in contemporary schools is English. Bengali students are frequently taught in English from secondary school, leading to widespread practical fluency and a habit of code-switching in social and academic contexts (Rahman, 2014; Hossain, 2016). Consequently, English acts as an academic common language and a practical tool for specific types of communication, influencing the bilingual skills of these individuals. Additionally, the participant group was drawn from a pre-existing, naturally assembled class, meaning the gender ratio reflects typical enrollment patterns rather than a deliberate sampling strategy.

They have been in China for about 3 months. Prior to traveling to China, the participants had not studied Chinese. Additionally, they have only recently arrived in China. As a result, their knowledge of Chinese culture and their proficiency in the language remain limited. Thus, the primary focus of this study is the linguistic settings of Bengali and English.

### 3.2 Video task

The participants were video recorded while doing the interviews in Bengali and English. Each participant participated in both sessions, separated by 4 weeks. Bengali session preceded English session for all participants. This ordering mirrors real-world language use: Bengali dominates home interactions earlier in the day, while English becomes primary for academic and professional activities later on. To guarantee the removal of memory effects and prevent any reciprocal influence between the two languages, there is a four-week gap between the two cycles. A four-week gap could minimize carry-over effects of cross-language structural priming (Gries and Kootstra, 2017) and satisfy ethical concerns about response bias. In addition, the participants spoke either Bengali or English throughout the entire round, and did not codeswitch between the two languages. While the English round lasts over 2.4 h (2.27 min for the shortest and 13.45 min for the longest), the Bengali round’s total video time is roughly 2.7 h, with 3 min for the shortest and 9.44 for the longest.

A bilingual student from Bangladesh was invited to serve as the host and initiate conversations with each participant in an open and quiet room. Since semi-structured interviews are typically based on a series of questions and mostly focus on a few key issues while also permitting probes, one was used (Karatsareas, 2022). Furthermore, unlike some carefully designed experiments (Cooperrider et al., 2018; Cooperrider and Núñez, 2024; Deichler et al., 2023), face-to-face communication was used to elicit pointing gestures in a more natural setting and minimize the use of technical equipment and human interventions, allowing participants to unconsciously display their true behaviors (Cooperrider, 2014). It improves the study’s ecological validity by allowing participants to make pointing movements in a more organic manner. Concurrently, the host started the interviews with warm-up chat, then followed the same semi-structured guide in both languages: (1) describing general learning experience, (2) highlighting the learning difficulties, (3) talking about the study facilities. The questions are arranged in fixed order but allow for flexible wording. Prompts like “what happened next?” or “how do you deal with that” are introduced to maintain cross-session consistency while preserving naturalness.

To maintain topical consistency across rounds, participants discussed their educational experiences in China because of its general familiarity. They were encouraged to speak comprehensively without time limits and were seated with hands free to gesture naturally. A front-facing camcorder on a tripod recorded them from 1.5 m (chest-up view), while a 45° side-angle camera captured hand movements. Shadow-free LED panels and armless chairs ensured full hand visibility, and continuous dual-camera recording preserved all gestures for coding.

### 3.3 Transcription

A reciprocal transcription method was implemented for the entire bilingual dataset by two fluent Bengali English speakers to achieve the highest possible accuracy. For both sessions, one transcriber created complete verbatim transcripts alongside English translations for all 30 recordings, including marking down the pauses during the talk. The other transcriber then performed an independent line-by-line review of the original audio, verified the translations, and identified any errors, inconsistencies, or timing issues. Disagreements were resolved by re-watching the video in question, discussing contextual subtleties, and collaboratively revising the transcript until a consensus was reached.

### 3.4 Gesture analysis

McNeill (1992) distinguished four types of hand gestures: iconic, metaphoric, deictic (pointing), and beat gestures. Iconic gestures mimic the shape, size, movement, or action described in the words; metaphoric gestures use concrete actions or objects to symbolize abstract concepts and ideas; deictic gestures help speakers and listeners navigate the shared space of the conversation by pointing to objects or locations in space; and beat gestures serve as rhythmic markers to emphasize speech, highlighting specific points or highlighting the speaker’s message.

Gesture coding was conducted by two independent coders who did not participate in audio transcription. The coders underwent 6 h of training led by one scholar familiar with the gesture coding, and they practiced the pilot videos until reaching 75%. McNeil’s gesture classification (McNeill, 1992) was adopted, focusing on specific gesture type such as deictics. Inter-rater reliability was assessed using Cohen’s Kappa, which revealed substantial agreement for hand pointing gestures in both the Bangladesh round (κ = 0.712, *p* < 0.001) and the English round (κ = 0.715, *p* < 0.001), and also non-hand pointing gestures in the Bangladesh round (κ = 0.779, *p* < 0.001) as well as the English round (κ = 0.717, *p* < 0.001). Discrepancies were resolved through discussion.

Deictic gestures, which identify objects, places, or directions, are frequently used in conjunction with speech to improve comprehension (Liebal et al., 2009). Pointing gestures are among the most common deictic gestures used in a variety of contexts and cultures (Kukier et al., 2025). Hand pointing frequently co-occurred with speech in both languages and was observed more often during the English sessions (total gesture rates: 85.19) compared to the Bengali sessions (total gesture rates: 42.01). Even while pointing is normally done by hand, certain cultures sometimes utilize non-manual ways, like head movements or facial expressions such as nose wrinkling or lip protrusion (Cooperrider and Mesh, 2022). The Bengali round (total gesture rates: 36.1) employs more non-hand gestures for pointing than the English round (total gesture rates: 24.09). At times, the participants simultaneously pointed with their hands and their non-hands; these were then captured and examined independently. Overall, the English round was dominated by manual pointing, while non-manual pointing was more prominent in the Bengali round.

Because the same participants contributed observations in both contexts, the data were paired rather than independent. Therefore, paired-sample *t* tests were performed using SPSS software to compare manual and non-manual pointing between the Bengali and English contexts, as this method is appropriate for within-participant comparisons and remains robust to moderate deviations from normality (Jennings et al., 2002).

## 4 Results

Preliminary study on the types of hand gestures shows that total gesture rates of deictic hand gestures in the Bengali round is 42.01, while it is 85.19 in the English round. From Table 2, there is significance for the deictic gesture rates in the two rounds (*t* = 3.41, df = 29, *p* < 0.05).

**Table 2:** Paired-samples *t*-tests comparing Bengali round vs. English round (gesture rate, *N* = 30)

Pointing form	Mean diff (E-B)	*t*	df	*p*	95% CI	Cohen’s dz
Manual pointing	1.44	3.41	29	0.002	[0.57, 2.30]	0.62
Non-manual pointing	−0.40	−3.43	29	0.002	[−0.64, −0.16]	−0.63

Further study explored the application of non-hand gestures for pointing in the two rounds. Besides using hand gestures for pointing, such non-hand gestures as head movements and facial expressions are also used for pointing. The gesture rates of non-hand gestures is 36.1 in the Bengali round, comparing with that in the English round (24.09). The significance does exist for the two rounds (*t* = −3.43, df = 29, *p* < 0.05) (see Table 2).

Moreover, as shown in Table 3, the manual pointing gesture rates in the English round (*M* = 2.84, SD = 1.97, median = 2.12, range = 0.71–7.97) was significantly higher than that in the Bengali round (*M* = 1.40, SD = 0.84, median = 1.37, range = 0–3.23). Conversely, the non-manual pointing frequency was significantly higher in the Bengali round (*M* = 1.20, SD = 0.49, median = 1.06, range = 0.24–2.42) than in the English round (*M* = 0.80, SD = 0.37, median = 0.73, range = 0.27–1.67).

**Table 3:** Descriptive statistics of manual and non-manual pointing gesture rate (*N* = 30).

Pointing form	Round	Gesture rate *M* (SD)	Median	Range
Manual pointing	Bengali	1.40 (0.84)	1.37	0.00–3.23
	English	2.84 (1.97)	2.12	0.71–7.97
Non-manual pointing	Bengali	1.20 (0.49)	1.06	0.24–2.42
	English	0.80 (0.37)	0.73	0.27–1.67

In summary, the results of the statistical analysis strongly support the proposed hypotheses. When participants spoke English, they elicited significantly more pointing gestures than when they speak Bengali, confirming H1, H3 that English high-text culture fosters explicit code for reference, thus more pointing gestures enhancing speech and lessening the cognitive load, whereas the richer Bengali input and low-context culture for shared understanding contribute to the lower use of pointing gestures in the Bengali context. In contrast, the high-context Bengali context produced almost twice as many non-hand points, supporting H2’s prediction that cultural norms of face-saving and indirectness would guide deictic reference toward more non-manual pointing such as head, eyes and lips movements. The language-specific re-distribution of this total pool reveals that bilinguals do not simply “point more” or “point less”; they reallocate the pointing load in response to the implicit cultural script cued by the language in use.

## 5 Discussion

This research examines manual and non-manual gestures used in Bengali and English settings. These gestures transcend simple physical motion; they operate as a cultural code, expressing politeness, social standing, and shared understanding. While communication in English tends to involve more hand gestures, Bengali often favors non-manual methods like head nods, lip pointing, or gaze direction to convey meaning. In line with H1, participants produced significantly more pointing gesture rates in English (85.19manual, 24.09 non-manual) than in Bengali (42.01manual, 36.1 non-manual); *t*-tests showed *p* < 0.05 for manual and *p* < 0.05 for non-manual differences. On the other hand, confirming H2 and H3, the use of non-manual pointing nearly doubled in Bengali, mirroring the culture’s high-context emphasis on indirect communication and maintaining social harmony. Taken together, manual acts outnumbered non-manual ones 127.2 to 60.19, but the critical pattern is the language-driven reallocation: English primed explicit, low-context strategies that prefer the hand pointing, whereas Bengali cued implicit, high-context routines that favor the non-hand pointing such as head or eyes movements.

Interestingly, no quantitative research has yet been conducted on gesture use among Bengali bilinguals. Existing gesture research has predominantly involved English speakers, with Bengali populations receiving little attention. This gap is particularly critical for pointing gestures. There are two key reasons this data is needed. First, Bengali is the world’s seventh most spoken language with about 284 million users (Visual Capitalist, 2025), but its communication style—featuring complex politeness systems, indirect cues, and prevalent non-manual pointing—contrasts greatly with the direct, finger-pointing common in English (Uddin, 2019; Tarek, 2017; Arif, 2011). Second, Bangladeshi diaspora communities are among the fastest-growing in several global regions (International Organization for Migration, 2025). Misinterpreting a lip point as evasive or a finger point as hostile can harm intercultural cooperation in schools and offices (Rahayu et al., 2025; Vohra, 2022). Without reliable evidence on how these bilinguals modify their pointing, professionals and technologies risk perpetuating misunderstandings that reinforce stereotypes and marginalize people.

The findings reveal that only deictic (pointing) and beat gestures showed significant variation across the two language contexts. Other gestures remained relatively stable. Deictic gestures allow speakers to specify spatial references. When speaking English, bilinguals produced 2.03 times as many pointing gesture rates in total as when speaking Bengali (85.19 vs. 42.01). This is not merely habitual but reflects the low-context nature of English communication, where explicit labeling of references is essential, and the hand becomes the most reliable tool. In contrast, Bengali culture is high-context, where shared knowledge and environmental cues suffice, making fewer hand gestures necessary. Moreover, just as speakers use pointing gestures to specify entities in the physical conversational context, they may similarly employ such gestures to relate to elements within the mentally simulated situation (Khatin-Zadeh et al., 2025b). The proportion of the concrete pointing gestures (e.g., participants said “whiteboard” and pointed at the whiteboard in the room) is 58%, whereas the rate of abstract pointing gestures (e.g., participants said “difficulty” and pointed upward) is 42%. These data disclose that the majority of pointing served concrete object reference instead of abstract functions.

A distinct pattern emerges for non-manual pointing. Table 2 indicates that gestures involving the head, lips, and eyes were far more common in Bengali than in English. This is because finger-pointing is frequently viewed as impolite in Bengali culture. Indicating direction with the head or lips is considered more respectful, particularly with elders or superiors. The use of these subtle pointers rises significantly in these social situations. Uddin (2019) notes that open-hand or head gestures often accompany formal language, whereas finger-pointing is used among close peers. Therefore, pointing in Bengali is not just a referential act but a social signal conveying respect and defining relationships. This finding is consistent with other cultures; the Yupno people, for instance, also favor non-manual pointing over the index-finger method typical of Americans (Cooperrider et al., 2018). This shows that non-manual pointing is not a crude method, but a sophisticated cultural tactic that values social cohesion over sheer efficiency. More importantly, the finding from Bengali preference for non-manual pointing provides a new perspective to understand cognitive diversity.

Studies demonstrate that bicultural individuals unconsciously shift between cultural frames in response to situational cues of cultural expectations (Aydinli and Bender, 2015). Cultural schemas can be primed without meaning-related cues. For instance, images, sounds, or even smells and tastes can trigger the particular cultural mind-sets (Leung and Morris, 2015). Cultural icons act as powerful symbols that can trigger associated ways of thinking and acting. In a seminal study, Hong et al. (2000) presented bicultural students with images like the White House or the Forbidden City; exposure to American icons led to more personality-focused attributions, while Chinese icons prompted more situationally based reasoning. Likewise, other research found that viewing a culturally significant object, such as a Chinese vase, made Chinese linguistic structures more cognitively accessible than English ones (Zhang et al., 2013).

The trigger for switching cultural frames can be something as fundamental as language choice. Simply using English activates a low-context mindset that demands explicit spatial reference. Within this frame, points in space must be clearly identified, as they are not assumed to be part of a shared understanding. The hands naturally become the primary instrument for this task due to their availability, mobility, and visual prominence. The manual gestures rates (127.2) vastly outnumber non-manual ones (60.19). This aligns with the observation that people invest more in manual gesturing when precision is required (Cooperrider et al., 2018). Thus, in the English mode, the hand is enlisted as the essential tool to fulfill the cultural need for clear and unambiguous spatial communication.

The frame shifts just as rapidly when switching to Bengali. A high-context script takes over, where maintaining social harmony becomes more important than pinpoint accuracy. In this mode, cultural norms counteract the general preference for manual pointing; a pointed finger is seen as rude, discouraging its use. The job of pointing is instead delegated to less socially risky body parts like the lips, eyes, and head, leading to a measurable rise in their use. Essentially, bilinguals adhere to an unspoken “polite-indirect” rule by employing these subtle signals, successfully indicating references while managing social expectations. The overall tendency to use hands is not eliminated but redistributed. They achieve clear reference and social grace simultaneously by directing the pointing function through whatever bodily means the active cultural context approves.

This research successfully incorporates pointing gestures into the CFS framework, demonstrating that language alone is a potent cultural trigger that can alter physical behavior. This extends previous work that relied mostly on visual primes. Moreover, it offers concrete physical evidence for high- and low-context cultural theories. The English setting, demanding explicit labels for space, results in more gestures overall. Conversely, the Bengali setting, activating norms of indirectness and politeness, causes a marked shift toward non-manual strategies. This proves that contextual contrasts are embodied and visibly expressed through gesture, not just through language.

This work addresses a gap in gesture research concerning whether variations in pointing are merely stylistic or tied to deeper cultural patterns. The results show that language itself is enough to cause immediate shifts between hand and non-hand pointing. It also pushes the CFS theory forward; whereas earlier studies used images and questionnaires (Qureshi, 2023; Garcha et al., 2025), this study confirms that language is a powerful and naturalistic cue that produces culturally congruent physical actions. This two-fold contribution solidifies the role of visible gesture in CFS and offers the field measurable, physical proof that bilinguals embody different cultural styles in real-time conversation.

## 6 Conclusion

By analyzing pointing gestures, this study reveals language’s role as a cultural trigger. Using English activates a communicative mode that values explicitness, leading to a rise in manual pointing. Using Bengali, however, activates a mode prioritizing indirectness and politeness, thereby increasing the use of non-manual pointing. During both sessions, the same 30 Bengali-English bilinguals produced 85.19 manual pointing gesture rates in English but only 42.01 in Bengali (*t* = 3.41, *p* < 0.05), while non-manual pointing gesture rates rose from 24.09 to 36.1 (*t* = −3.43, *p* < 0.05). Functioning as a cultural trigger, language reallocates how pointing is performed, providing measurable, physical proof that bilinguals dynamically switch between high-context and low-context cultural frames as they speak.

A limitation of this study is its participant pool, which consisted solely of Bengali international students from one Chinese university. Future work should aim to include bilinguals from a wider range of professions and age groups. Also, given that gender can affect how and how often people gesture (Gallo et al., 2024), the current results might not be fully generalizable to female Bengali-English bilinguals. Future studies should strive for a more balanced sample to investigate potential gender-based variations in gesture use. Moreover, another stream of future research is to deconstruct the pointing gestures generated by the head, eyes, nose, chin and other sub-movements within the “non-hand” category, and work out the specific social symbols and politeness values they carry in the Bengali cultural context, therefore enhancing the explanation of functional differences among non-hand gestures.

The gesture switches between these two cultural frames flexibly, disclosing that communication is not only a verbal contract but an embodied, context-situated cognitive process. These insights can help prevent cross-cultural misunderstandings and improve interaction in Bengali-English contexts. They can also inform technology design by enabling AI and robots to recognize non-manual pointing. On a broader scale, these findings enrich our understanding of the bilingual mind and open new avenues for exploring the fundamental elements of human interaction.

## Data Availability

The raw data supporting the conclusions of this article will be made available by the authors, without undue reservation.
